# Does acute maternal stress in pregnancy affect infant health outcomes? Examination of a large cohort of infants born after the terrorist attacks of September 11, 2001

**DOI:** 10.1186/1471-2458-9-252

**Published:** 2009-07-20

**Authors:** Skye M Endara, Margaret AK Ryan, Carter J Sevick, Ava Marie S Conlin, Caroline A Macera, Tyler C Smith

**Affiliations:** 1 US Department of Defense Center for Deployment Health Research, Naval Health Research Center, San Diego, CA, USA; 2 Naval Hospital Camp Pendleton, Camp Pendleton, CA, USA; 3 San Diego State University, Graduate School of Public Health, Division of Epidemiology and Biostatistics, San Diego, CA, USA

## Abstract

**Background:**

Infants in utero during the terrorist attacks of September 11, 2001 may have been negatively affected by maternal stress. Studies to date have produced contradictory results.

**Methods:**

Data for this retrospective cohort study were obtained from the Department of Defense Birth and Infant Health Registry and included up to 164,743 infants born to active-duty military families. Infants were considered exposed if they were in utero on September 11, 2001, while the referent group included infants gestating in the same period in the preceding and following year (2000 and 2002). We investigated the association of this acute stress during pregnancy with the infant health outcomes of male:female sex ratio, birth defects, preterm birth, and growth deficiencies in utero and in infancy.

**Results:**

No difference in sex ratio was observed between infants in utero in the first trimester of pregnancy on September 11, 2001 and infants in the referent population. Examination of the relationship between first-trimester exposure and birth defects also revealed no significant associations. In adjusted multivariable models, neither preterm birth nor growth deficiencies were significantly associated with the maternal exposure to the stress of September 11 during pregnancy.

**Conclusion:**

The findings from this large population-based study suggest that women who were pregnant during the terrorist attacks of September 11, 2001 had no increased risk of adverse infant health outcomes.

## Background

On September 11, 2001, the United States suffered terrorist attacks against the World Trade Center and the Pentagon. The psychological stress resulting from these events affected not only those individuals who witnessed or experienced them directly, but people across the nation as well [1-7]. Studies have shown high levels of posttraumatic stress symptoms in women following the attacks [1,7]. Moreover, pregnant women suffering psychological sequelae from the attacks are of particular interest because the psychological well-being of a woman during pregnancy may affect infant health outcomes [8-10].

Biological studies of stress hormones suggest that maternal stress may be associated with the timing of parturition [11-14]. High levels of the corticotropin-releasing hormone (CRH), a key component of the stress response system, have been observed in women delivering preterm [13,14]. In addition, CRH is associated with fetal growth restriction [13].

In New York and California, reproductive health effects, such as reductions in birth sex ratios (an indirect measure of pregnancy loss), have been associated with acute maternal stress after the events of September 11 [15,16]. However, other studies using populations within and outside New York City have found contradicting results. For example, in New York, an analysis from in vitro fertilization clinics noted a significant increase in pregnancy losses and a reduced delivery rate among women whose pregnancy tests occurred after the terrorist attacks [17]. Another study in New York observed intrauterine growth restriction in infants in utero during the terrorist attacks, but no significant differences were found in mean gestational age or birthweight [18]. Furthermore, shortened gestation was observed among women living in proximity to the World Trade Center [19], while other studies in the area revealed longer gestation [20] or a decrease in preterm births [21]. All of the aforementioned studies, except Eskenazi *et al*. (2007), were limited by relatively small (fewer than 300 participants) sample sizes [17-20]. Pregnant women living near the World Trade Center were exposed to significant amounts of air pollution [22,23]. Thus, it is not clear whether environmental factors such as air quality were largely responsible for results seen in New York populations. Studies conducted outside of New York may have been able to exclude the effects air pollution may have had on the fetus [24,25].

Mixed results regarding the events of September 11 and several birth outcomes may be reconciled, in part, by larger population-based analyses. The Department of Defense (DoD) Birth and Infant Health Registry includes births from all 50 states, the District of Columbia, and more than 20 foreign countries [26]. As such, this data source will provide insight on stress and pregnancy following September 11 from a large population. The purpose of this retrospective cohort analysis following September 11 was to explore the association of this acute stress during pregnancy with sex differences, the prevalence of birth defects, preterm birth, and growth deficiencies in utero and in infancy.

## Methods

### Population

The population for this study included up to 164,743 infants born to active-duty military families included in the DoD Birth and Infant Health Registry. Specifically, the cohort of infants "exposed" to the stress of September 11 during pregnancy was compared with a referent population composed of all infants born to US military families whose gestations were exactly comparable in timing from 2000 and 2002. Approximately 19% of infants in the database were born to women serving in the military; others were born to families in which the father was serving in the military [26].

### Data sources

The primary data source for this study was the DoD Birth and Infant Health Registry [26]. Since 1998, the Registry has assembled and validated birth and health information about infants born to US military families [26]. This database provided information on estimated gestational age (EGA) at birth, sex, and all medical diagnoses from birth through the first year of life. The DoD Birth and Infant Health Registry captures inpatient and outpatient health care data, from both military and civilian facilities, on all infants born to military beneficiaries. Registry data samples have been validated for gestational age, birth defects, and infant neoplasm diagnoses [26]. The use of Registry data for this study has been approved by the Naval Health Research Center Institutional Review Board, under protocol number NHRC.2008.0015.

Demographic and military personnel data include maternal age (categorized in groups younger than 35 years, and 35 years and older), maternal military status (civilian or active duty), infant's military sponsor's education level (high school or less, some college, or bachelor's degree or higher), race/ethnicity (white non-Hispanic, black non-Hispanic, Hispanic, Asian/Pacific Islander, other), service branch (Army, Navy/Coast Guard, Air Force or Marine Corps), pay grade (enlisted or officer), and occupation (combat specialists, health care specialists, or other).

### Outcome definitions

Infant sex was evaluated from birth records. Male:female sex ratio was calculated among infants born to women who experienced the stress of September 11 during their first trimester of pregnancy (0–13 weeks EGA), and compared with the referent population.

Major birth defects were evaluated using the definitions from the National Birth Defects Prevention Network [27], which included a subset of *International Classification of Diseases 9th Revision, Clinical Modification *(ICD-9-CM)[28] 4- and 5-digit codes from 740.x-760.x. Cases of atrial septal defect (745.5x) and patent ductus arteriosus (747.0x) in preterm infants were not included as birth defects in accordance with Metropolitan Atlanta Congenital Defects Program guidelines [29]. Health care use records through the 12-month period after birth were assessed to capture birth defects diagnosed in infancy.

Preterm birth was assessed using EGA, defined by ICD-9-CM codes for weeks of gestation, 765.0x-765.2x. Due to ICD-9-CM limitations, an infant's EGA at birth was assigned using the maximum end of each range. Infants born with less than 37 completed weeks of gestation were considered preterm. If more than one 5-digit code existed for a single infant, as might occur if EGA was revised over time, the most recent code assigned was used. Additionally, multiple births were excluded from this analysis, since multiple gestations would confound the evaluation of preterm delivery.

Growth deficiencies in utero were examined using ICD-9-CM codes for slow fetal growth and fetal malnutrition (764.x), often clinically referred to as "small-for-gestational age" (SGA). These codes may be in the birth record, while codes for growth deficiencies in infancy may appear on records from birth through the first year of life. Growth deficiencies in infancy were examined using ICD-9-CM codes indicating lack of normal physiological development in childhood (783.4x). Code 783.42 (delayed milestones-late walker, late talker) was not included as a growth deficiency in infancy, since this code is not expected to be applied during the first year of life.

### Defining exposure and referent populations

The terrorist attacks of September 11, 2001 were considered a universal acute stressor. For analyses of male:female infant sex ratio and birth defects, exposed infants were defined as those born to women in their first trimester of pregnancy on September 11. The first trimester of pregnancy was considered 13 weeks after the onset of pregnancy, and was selected because this is the period of greatest vulnerability to teratogens or fetal compromise [30], and for comparability with other studies. Onset of pregnancy is clinically defined as the first day of the ovulatory cycle in which the pregnancy was conceived; this may correspond to the first day of the last menstrual period before conception in women with regular menstrual cycles. Mathematically, onset of pregnancy is calculated as exactly 40 weeks (280 days) before the estimated date of delivery of a full-term pregnancy. Onset of pregnancy may also be called day zero of the estimated gestational age (EGA zero). For this analysis, therefore, exposed infants had EGA zero from 13 weeks (91 days) prior to September 11, 2001 (i.e., June 12, 2001) through EGA zero of September 11, 2001.

The referent group of infants for analyses of sex ratio and birth defects included those conceived exactly 1 year prior and 1 year after the exposed group. Specifically, the referent group included all infants with EGA zero between June 12, 2000 and September 11, 2000, and those with EGA zero between June 12, 2002 and September 11, 2002.

The selection of a referent group that included conceptions 1 year prior and 1 year after the exposure of interest was intended to control for both seasonal variations and annual temporal trends in the outcomes of interest. It is possible, however, that infants conceived in 2002 would still be "exposed" to the stress of September 11, 2001 due to long-lasting sequelae from the attacks. Therefore, alternative analyses limited the referent group to only those infants conceived 1 year prior to the exposed group.

To examine the outcomes of preterm birth and growth deficiencies in utero and in infancy, the exposed group of infants was expanded to include all those born to women who were pregnant, but less than full term, on September 11, 2001. Thus, the exposure group for these outcomes includes infants in utero, from 0–36 weeks EGA, on September 11, 2001. Full term may be clinically defined as pregnancy later than 37 completed weeks (252 days) EGA. Thus, the exposed group of infants in these analyses had EGA zero from January 2, 2001 to September 11, 2001, and were born after September 11, 2001.

Consistent with the definitions above, the referent group of infants for analyses of preterm birth and growth problems included those conceived exactly 1 year before and 1 year after the exposed group. Specifically, the referent group had EGA zero from January 2, 2000 to September 11, 2000, and were born after September 11, 2000; or had EGA zero from January 2, 2002 to September 11, 2002, and were born after September 11, 2002. As above, alternative analyses limited the referent group to only those infants conceived 1 year prior to the exposed group.

### Statistical analyses

Univariate analyses, including chi-square tests, were conducted to determine significant associations between infant health outcomes and maternal exposure to the stressors of September 11 during pregnancy. Multivariable logistic regression analyses were completed, adjusting models for maternal age and other demographic variables. Regression diagnostics were applied. Presence of multicollinearity was assessed using the variance inflation factor, with a value greater than 4 suggesting presence of this condition. No variables in the models used met the threshold for multicollinearity. Adjusted odds of health outcomes of infants born to active-duty military families exposed in utero to the acute stress of September 11 were compared with infants born to active-duty military families in the referent population. Data management and statistical analyses were performed using SAS software version 9.1.3 (SAS Institute, Inc., Cary, NC, USA).

## Results

### Infants in utero, from 0 to 13 weeks EGA, on September 11, 2001

**Figure 1:**
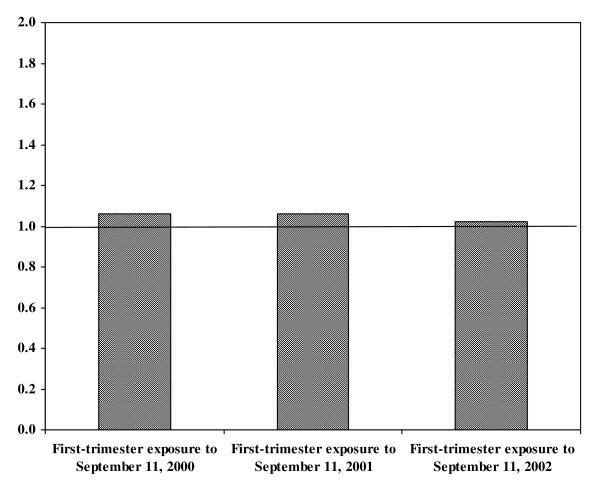
**Male:female sex ratio of infants born to military families**. Based on exposure to September 11, 2001 and referent populations, the number of male infants born to US military families divided by the number of females born to US military families.

Male:female infant sex ratio and birth defects were evaluated based on exposure to the stressors of September 11, 2001 in first-trimester pregnancy, with referent groups in the first trimester of pregnancy in years 2000 and 2002. Of 61,088 infants born to military families in this timeframe, 49% (29,841) were female and 51% (31,247) were male. No differences in the male:female sex ratios of infants were observed among the 2000, 2001, and 2002 infant cohorts (Figure 1). In addition, multivariable logistic regression did not reveal a significant association between maternal stress from the terrorist attacks and the sex of an infant (OR 1.02, 95% CI [0.99, 1.06]).

Infants with at least 1 major birth defect, defined by ICD-9-CM coding in the first year of life, accounted for 4.9% (3,005) of the study population. Table 1 shows the results of an adjusted multivariable logistic regression model for birth defects. Covariates included maternal age, maternal military status, military parent's education, race/ethnicity, service branch, pay grade, and occupation. Infants with birth defects were not proportionally different from infants without birth defects (*N *= 58,083), with respect to most parental demographic variables. Infants born to women of advanced maternal age (≥ 35 years) had a significantly higher prevalence of birth defects (odds ratio [OR] 1.21, 95% confidence interval [CI; 1.06, 1.38]) compared with infants born to younger women. No significant association was found between infants in utero, from 0–13 weeks EGA, on September 11, 2001, and birth defects, relative to infants in the referent population (OR 1.01, 95% CI [0.93, 1.09]).

**Table 1:** Adjusted odds^a ^of birth defects among US military family infants born after in utero exposure to stress

Characteristic	All infants n = 61088		Infants with birth defects^b^ n = 3005		OR [95% CI]
	n	(%)	n	(%)	
In utero exposure to September 11, 2001^c^					
No	41272	(67.6)	2026	(67.4)	1.00
Yes	19816	(32.4)	979	(32.6)	1.01 [0.93, 1.09]
Maternal age					
<35 years	56205	(92.0)	2721	(90.5)	1.00
≥ 35 years	4883	(8.0)	284	(9.5)	1.21 [1.06, 1.38]
Maternal military status					
Civilian/married to active duty	49660	(81.3)	2484	(82.7)	1.00
Active duty	11428	(18.7)	521	(17.3)	0.95 [0.86, 1.05]
Military parent's education level					
Bachelor's or above	11326	(18.5)	561	(18.7)	1.00
Some college	2478	(4.1)	152	(5.1)	1.24 [0.97, 1.59]
High school or less	47284	(77.4)	2292	(76.3)	0.98 [0.81, 1.20]
Military parent's race/ethnicity					
White/non-Hispanic	40386	(66.1)	2096	(69.8)	1.00
Black/non-Hispanic	11017	(18.0)	497	(16.5)	0.87 [0.79, 0.97]
Hispanic	5993	(9.8)	271	(9.0)	0.87 [0.76, 0.99]
Asian/Pacific Islander	2294	(3.8)	83	(2.8)	0.69 [0.55, 0.86]
Other	1398	(2.3)	58	(1.9)	0.80 [0.61, 1.04]
Military parent's service branch					
Army	23010	(37.7)	1100	(36.6)	1.00
Navy/Coast Guard	15012	(24.6)	735	(24.5)	1.03 [0.94, 1.14]
Air Force	15859	(26.0)	786	(26.2)	1.00 [0.91, 1.10]
Marine Corps	7207	(11.8)	384	(12.8)	1.11 [0.98, 1.25]
Military parent's pay grade					
Officer	10363	(17.0)	515	(17.1)	1.00
Enlisted	50725	(83.0)	2490	(82.9)	1.05 [0.85, 1.29]
Military parent's occupation					
All others	42699	(69.9)	2118	(70.5)	1.00
Health care specialists	5294	(8.7)	236	(7.9)	0.92 [0.80, 1.06]
Combat specialists	13095	(21.4)	651	(21.7)	0.99 [0.90, 1.09]

^a^Based on multivariable logistic regression.

^b^Evaluated using criteria from the National Birth Defects Prevention Network.

^c^Exposed infants were in utero from 0 to 13 weeks EGA on September 11, 2001.

Unexposed infants were in utero from 0 to 13 weeks EGA on September 11, 2000, or September 11, 2002.

OR, odds ratio; CI, confidence interval.

### Infants in utero, from 0 to 36 weeks EGA, on September 11, 2001

Preterm birth, growth deficiencies in utero, and growth deficiencies in infancy, were evaluated based on exposure from 0 to 36 weeks EGA, on September 11, 2001, with referent groups in the same time interval in 2000 and 2002. There were 164,743 infants who met these criteria and were included in these analyses.

Approximately 7% of infants in this population were born preterm. Of these 11,453 infants, 3,761 were in utero on September 11, 2001. Table 2 shows demographic characteristics as well as the results from multivariable logistic regression. Infants born to women of advanced maternal age, infants born to parents of black non-Hispanic race/ethnicity, and infants born to parents in enlisted pay grades had slightly increased odds of preterm birth. Adjusting for maternal age and all parental demographic variables, there was no statistically significant association between in utero exposure to September 11 and preterm birth (OR 1.02, 95% CI [0.98,1.06]).

**Table 2:** Adjusted odds^a ^of preterm birth among US military family infants born after in utero exposure to stress

Characteristic	All infants n = 164743		Infants born preterm^b^ n = 11453		OR [95% CI]
	n	%	n	%	
In utero exposure to September 11, 2001^c^					
No	111413	(67.6)	7692	(67.2)	1.00
Yes	53330	(32.4)	3761	(32.8)	1.02 [0.98,1.06]
Maternal age					
<35 years	151571	(92.0)	10429	(91.1)	1.00
≥ 35 years	13172	(8.0)	1024	(8.9)	1.22 [1.14, 1.31]
Maternal military status					
Civilian/married to active duty	133152	(80.8)	9125	(79.7)	1.00
Active duty	31591	(19.2)	2328	(20.3)	1.02 [0.97, 1.07]
Military parent's education level					
Bachelor's or above	30097	(18.3)	1761	(15.4)	1.00
Some college	6715	(4.1)	467	(4.1)	1.09 [0.95, 1.25]
High school or less	127931	(77.7)	9225	(80.5)	1.10 [0.99, 1.23]
Military parent's race/ethnicity					
White/non-Hispanic	108252	(65.7)	7172	(62.6)	1.00
Black/non-Hispanic	30300	(18.4)	2670	(23.3)	1.30 [1.24, 1.37]
Hispanic	16097	(9.8)	985	(8.6)	0.88 [0.82, 0.95]
Asian/Pacific Islander	6183	(3.8)	388	(3.4)	0.91 [0.82, 1.02]
Other	3911	(2.4)	238	(2.1)	0.89 [0.77, 1.01]
Military parent's service branch					
Army	61822	(37.5)	4412	(38.5)	1.00
Navy/Coast Guard	41294	(25.1)	3031	(26.5)	1.03 [0.99, 1.09]
Air Force	42103	(25.6)	2630	(23.0)	0.89 [0.85, 0.94]
Marine Corps	19524	(11.9)	1380	(12.0)	1.01 [0.95, 1.08]
Military parent's pay grade					
Officer	27752	(16.8)	1601	(14.0)	1.00
Enlisted	136991	(83.2)	9852	(86.0)	1.14 [1.02, 1.27]
Military parent's occupation					
All others	115269	(70.0)	8150	(71.2)	1.00
Health care specialists	14366	(8.7)	985	(8.6)	1.01 [0.94,1.03]
Combat specialists	35108	(21.3)	2318	(20.2)	0.98 [0.94,1.04]

^a^Based on multivariable logistic regression.

^b ^Defined as estimated gestational age (EGA) less than 37 completed weeks.

^c^Exposed infants were in utero from 0 to 36 weeks EGA on September 11, 2001.

Unexposed infants were in utero from 0 to 36 weeks EGA on September 11, 2000 or September 11, 2002.

OR, odds ratio; CI, confidence interval.

The logistic regression models for growth deficiencies in utero and growth deficiencies in infancy adjusted for the same covariates as the previous models (Table 3). There were 828 (1.6%) infants in utero on September 11, 2001 who were diagnosed with a growth deficiency in utero. There were 2,073 (3.9%) exposed infants who were diagnosed with a growth deficiency in infancy. Adjusting for all other variables in the model, no statistically significant associations were observed between exposure to the stressors of September 11, 2001, and growth deficiencies in utero (OR 1.00, 95% CI [0.92, 1.09]). Moreover, there was no significant association observed between exposure to September 11 and growth deficiencies in infancy (OR 1.00, 95% CI [0.95, 1.06]).

**Table 3:** Adjusted odds^a ^of growth deficiencies among US military family infants born after in utero exposure to stress

Characteristic	All infants n = 164743		Infants with growth deficiencies in utero^b^ n = 2551			Infants with growth deficiencies in infancy^c^ n = 6407		
	n	(%)	n	(%)	OR [95% CI]	n	(%)	OR [95% CI]
In utero exposure to September 11, 2001^d^								
No	111413	(67.6)	1723	(67.5)	1.00	4334	(67.6)	1.00
Yes	53330	(32.4)	828	(32.5)	1.00 [0.92, 1.09]	2073	(32.4)	1.00 [0.95, 1.06]
Maternal age								
<35 years	151571	(92.0)	2364	(92.7)	1.00	5779	(90.2)	1.00
≥ 35 years	13172	(8.0)	187	(7.3)	1.00 [0.85, 1.16]	628	(9.8)	1.21 [1.11, 1.32]
Maternal military status								
Civilian/married to active duty	133152	(80.8)	1904	(74.6)	1.00	5581	(87.1)	1.00
Active duty	31591	(19.2)	647	(25.4)	1.27 [1.15, 1.40]	826	(12.9)	0.65 [0.61, 0.71]
Military parent's education level								
Bachelor's or above	30097	(18.3)	355	(13.9)	1.00	1346	(21.0)	1.00
Some college	6715	(4.1)	85	(3.3)	0.95 [0.70, 1.28]	251	(3.9)	0.92 [0.77, 1.10]
High school or less	127931	(77.7)	2111	(82.8)	1.15 [0.91, 1.45]	4810	(75.1)	0.96 [0.84, 1.10]
Military parent's race/ethnicity								
White/non-Hispanic	108252	(65.7)	1384	(54.3)	1.00	4570	(71.3)	1.00
Black/non-Hispanic	30300	(18.4)	704	(27.6)	1.69 [1.53, 1.86]	988	(15.4)	0.84 [0.78, 0.90]
Hispanic	16097	(9.8)	259	(10.2)	1.19 [1.04, 1.36]	512	(8.0)	0.77 [0.70, 0.85]
Asian/Pacific Islander	6183	(3.8)	137	(5.4)	1.65 [1.38, 1.97]	211	(3.3)	0.84 [0.73, 0.97]
Other	3911	(2.4)	67	(2.6)	1.28 [1.00, 1.64]	126	(2.0)	0.79 [0.66, 0.95]
Military parent's service branch								
Army	61822	(37.5)	927	(36.3)	1.00	2452	(38.3)	1.00
Navy/Coast Guard	41294	(25.1)	759	(29.8)	1.23 [1.11, 1.35]	1482	(23.1)	0.91 [0.85, 0.97]
Air Force	42103	(25.6)	574	(22.5)	0.98 [0.88, 1.09]	1614	(25.2)	0.94 [0.88,1.01]
Marine Corps	19524	(11.9)	291	(11.4)	1.06 [0.92, 1.21]	859	(13.4)	1.08 [1.00,1.17]
Military parent's pay grade								
Officer	27752	(16.8)	320	(12.5)	1.00	1256	(19.6)	1.00
Enlisted	136991	(83.2)	2231	(87.5)	1.09 [0.86, 1.39]	5151	(80.4)	0.94 [0.82, 1.09]
Military parent's occupation								
All others	115269	(70.0)	1828	(71.7)	1.00	4412	(68.9)	1.00
Health care specialists	14366	(8.7)	236	(9.3)	1.02 [0.89, 1.18]	502	(7.8)	0.97 [0.88, 1.07]
Combat specialists	35108	(21.3)	487	(19.1)	1.01 [0.91, 1.12]	1493	(23.3)	1.01 [0.95, 1.07]

^a^Based on multivariable logistic regression.

^b^Defined using ICD-9-CM codes 764.0, 764.1, 764.2, and 764.9.

^c^Defined using ICD-9-CM codes 783.40, 783.41, 783.43, and 783.4.

^d^Exposed infants were in utero from 0 to 36 weeks estimated gestational age (EGA) on September 11, 2001. Unexposed infants were in utero from 0 to 36 weeks EGA on September 11, 2000, or September 11, 2002.

OR, odds ratio; CI, confidence interval.

In alternative models, infants in utero from 0 to 13 weeks EGA, on September 11, 2000, were considered the referent group for the analysis of birth defects and sex ratio. Infants in utero, from 0 to 36 weeks EGA, on September 11, 2000, were considered the referent group for analyses of preterm birth, growth deficiencies in utero, and growth deficiencies in infancy. No statistically significant associations were observed between maternal exposure to September 11 and birth defects, preterm birth, and growth deficiencies in utero or in infancy. Detailed results from the alternative analyses are not shown.

## Discussion

In this study, we analyzed specific health outcomes of infants in utero during the terrorist attacks of September 11, 2001, and compared them with infants in utero in the preceding and following years (2000 and 2002). We found no significant associations between exposure to this putative maternal stressor and adverse outcomes. Specifically, infants born to exposed women had the same sex ratio, prevalence of birth defects, prevalence of preterm births, and prevalence of growth deficiencies as infants born to the referent population. These findings were consistent when alternative referent populations were considered.

Using the terrorist attacks of September 11, 2001 as a crude measure of acute maternal stress, we hypothesized that birth defects and the male:female sex ratio might be different among infants exposed to such acute maternal stress in the first trimester of pregnancy. Prior studies suggest that sex ratios may differ after maternal exposure to September 11, 2001 [15,16]. Exposure was restricted to the first trimester in studying these outcomes because the early gestational weeks represent the period of greatest vulnerability to teratogens or fetal compromise resulting in pregnancy loss [30]. Evaluating incident pregnancy loss was not possible with existing data for this study. However, lower male:female sex ratios among liveborn infants may be an indirect indicator of excessive pregnancy losses in a population [31-35]. We found no evidence that first-trimester exposure to September 11, 2001 was associated with these outcomes.

We further hypothesized that the prevalence of preterm births and growth deficiencies in utero and growth deficiencies in infancy might be different among infants exposed to the maternal stress of September 11, 2001 at any time during pregnancy. Exposure at any time in the gestational period, up to full-term EGA, was considered when evaluating these outcomes because such exposures could plausibly affect preterm birth, fetal growth, or infant growth [36]. Again, we found no significant associations in these models. Our results may help reconcile some of the conflicting results on infant health outcomes after September 11 reported from other populations [18-21,24,25].

Although our findings may be applicable to the general population, the possibility exists that military families experienced the stress of September 11, 2001 in unique ways. One study has suggested that military members experienced healthy psychological responses in the months after the terrorist attacks [37]. The authors hypothesized that this may have been due to an outpouring of national support for the military and first-responders, resulting in high job satisfaction and sense of purpose. Military families, however, may have had increased stress due to the chance of their loved ones being mobilized as first responders [38] and/or being deployed overseas [39].

The effects of acute maternal stress on pregnancy outcomes are complex. Most prior studies following natural disasters, such as earthquakes and nationally stressful events (e.g., assassination of politicians), suggest that maternal stress plays a role in birth outcome [40,41]. Similar to our analyses, most studies have used objective measures for adverse outcomes, such as preterm birth. Considering subjective outcomes in hypotheses about maternal stress, however, may be important as well. For example, mothers with posttraumatic stress disorder have reported that their infants had greater distress to the unfamiliar than mothers not suffering posttraumatic stress [8]. This may be important for future research.

Defining acute maternal stress based on the date of September 11, 2001 makes several assumptions about the event, since the stress was experienced differently by many individuals. This study cannot account for self-reported perception or response to the stressful event on an individual basis, and could contribute to the lack of significant findings in this study. Nonetheless, the objective measure of a catastrophic event limits biases related to recall and response. Other limitations of our analyses may relate to use of health care databases to define EGA and ICD-9-CM-code outcomes. Although validated on several measures, health care databases contain a margin of error that can influence results. Any misclassification bias resulting from the use of these databases was likely to be nondifferential. Finally, military databases, while providing extensive demographic information, are vulnerable to some unique limitations. In particular, births to both married and unmarried military women are identified in the DoD Birth and Infant Health Registry, but only births to married military men are identified since partners of unmarried men are not military beneficiaries. Also, distinguishing births to dual-military parents was not possible, since only one parent can be identified as the military sponsor of a beneficiary child in the databases. Defining the exposed and referent groups from the same population, however, should have mitigated any challenges related to such idiosyncrasies.

Despite these limitations, the analyses contain valuable information. The DoD Birth and Infant Health Registry is well positioned to study birth defects, preterm births, and growth problems in utero or in infancy because the database contains all available medical diagnoses during the first year of life. This information is especially important for birth defects diagnoses that may present weeks or months after delivery. In addition, the large sample size of geographically diverse infants makes the detection of rare events possible.

## Conclusion

In summary, findings from this large population-based study suggest that women who were pregnant during the terrorist attacks of September 11, 2001 had no increased risk of adverse infant health outcomes. Future analyses, such as those planned using prospective data from the Millennium Cohort [42], remain important for evaluating the effects of other maternal and paternal stressors on long-term reproductive health.

## Abbreviations

CRH: corticotropin-releasing hormone; DoD: The Department of Defense; ICD-9-CM: International Classification of Diseases 9th Revision, Clinical Modification; EGA: estimated gestational age: SGA: small for gestational age.

## Competing interests

The authors declare that they have no competing interests.

## Authors' contributions

SME, MAKR, CJS, ASC, CAM and TCS were responsible for the design, planning, analysis, writing, and revising of the manuscript. All authors read and approved the final manuscript.

## Pre-publication history

The pre-publication history for this paper can be accessed here:



## References

[B1] Pulcino T, Galea S, Ahern J, Resnick H, Foley M, Vlahov D (2003). Posttraumatic stress in women after the September 11 terrorist attacks in New York City. J Womens Health (Larchmt).

[B2] Galea S, Ahern J, Resnick H, Kilpatrick D, Bucuvalas M, Gold J, Vlahov D (2002). Psychological sequelae of the September 11 terrorist attacks in New York City. N Engl J Med.

[B3] Schuster MA, Stein BD, Jaycox L, Collins RL, Marshall GN, Elliott MN, Zhou AJ, Kanouse DE, Morrison JL, Berry SH (2001). A national survey of stress reactions after the September 11, terrorist attacks. N Engl J Med.

[B4] DeLisi LE, Maurizio A, Yost M, Papparozzi CF, Fulchino C, Katz CL, Altesman J, Biel M, Lee J, Stevens P (2001). A survey of New Yorkers after the Sept. 11, 2001, terrorist attacks. Am J Psychiatry.

[B5] Marshall RD, Galea S (2004). Science for the community: assessing mental health after 9/11. J Clin Psychiatry.

[B6] Stein BD, Elliott MN, Jaycox LH, Collins RL, Berry SH, Klein DJ, Schuster MA (2004). A national longitudinal study of the psychological consequences of the September 11, 2001 terrorist attacks: reactions, impairment, and help-seeking. Psychiatry.

[B7] Silver RC, Holman EA, McIntosh DN, Poulin M, Gil-Rivas V (2002). Nationwide longitudinal study of psychological responses to September 11. JAMA.

[B8] Brand SR, Engel SM, Canfield RL, Yehuda R (2006). The effect of maternal PTSD following in utero trauma exposure on behavior and temperament in the 9-month-old infant. Ann N Y Acad Sci.

[B9] Yehuda R, Engel SM, Brand SR, Seckl J, Marcus SM, Berkowitz GS (2005). Transgenerational effects of posttraumatic stress disorder in babies of mothers exposed to the World Trade Center attacks during pregnancy. J Clin Endocrinol Metab.

[B10] Obel C, Henriksen TB, Secher NJ, Eskenazi B, Hedegaard M (2007). Psychological distress during early gestation and offspring sex ratio. Hum Reprod.

[B11] Mulder EJ, Robles de Medina PG, Huizink AC, Bergh BR Van den, Buitelaar JK, Visser GH (2002). Prenatal maternal stress: effects on pregnancy and the (unborn) child. Early Hum Dev.

[B12] Hobel CJ (2004). Stress and preterm birth. Clinical Obstetrics and Gynecology.

[B13] Wadhwa PD, Garite TJ, Porto M, Glynn L, Chicz-DeMet A, Dunkel-Schetter C, Sandman CA (2004). Placental corticotropin-releasing hormone (CRH), spontaneous preterm birth, and fetal growth restriction: a prospective investigation. Am J Obstet Gynecol.

[B14] Mancuso RA, Schetter CD, Rini CM, Roesch SC, Hobel CJ (2004). Maternal prenatal anxiety and corticotropin-releasing hormone associated with timing of delivery. Psychosomatic Medicine.

[B15] Catalano R, Bruckner T, Gould J, Eskenazi B, Anderson E (2005). Sex ratios in California following the terrorist attacks of September 11, 2001. Hum Reprod.

[B16] Catalano R, Bruckner T, Marks AR, Eskenazi B (2006). Exogenous shocks to the human sex ratio: the case of September 11, 2001 in New York City. Hum Reprod.

[B17] Spandorfer S, Grill E, Davis O, Fasouliotis S, Rosenwaks Z (2003). September 11th in New York City (NYC): the effect of a catastrophe on IVF outcome in a New York City based program. Fertility and Sterility.

[B18] Berkowitz GS, Wolff MS, Janevic TM, Holzman IR, Yehuda R, Landrigan PJ (2003). The World Trade Center disaster and intrauterine growth restriction. JAMA.

[B19] Lederman SA, Rauh V, Weiss L, Stein JL, Hoepner LA, Becker M, Perera FP (2004). The effects of the World Trade Center event on birth outcomes among term deliveries at three lower Manhattan hospitals. Environ Health Perspect.

[B20] Engel SM, Berkowitz GS, Wolff MS, Yehuda R (2005). Psychological trauma associated with the World Trade Center attacks and its effect on pregnancy outcome. Paediatr Perinat Epidemiol.

[B21] Eskenazi B, Marks AR, Catalano R, Bruckner T, Toniolo PG (2007). Low birthweight in New York city and upstate New York following the events of September 11th. Hum Reprod.

[B22] Wolff MS, Teitelbaum SL, Lioy PJ, Santella RM, Wang RY, Jones RL, Caldwell KL, Sjodin A, Turner WE, Li W (2005). Exposures among pregnant women near the World Trade Center site on 11 September 2001. Environ Health Perspect.

[B23] Perera FP, Tang D, Rauh V, Lester K, Tsai WY, Tu YH, Weiss L, Hoepner L, King J, Del Priore G (2005). Relationships among polycyclic aromatic hydrocarbon-DNA adducts, proximity to the World Trade Center, and effects on fetal growth. Environ Health Perspect.

[B24] Smits L, Krabbendam L, de Bie R, Essed G, van Os J (2006). Lower birth weight of Dutch neonates who were in utero at the time of the 9/11 attacks. J Psychosom Res.

[B25] Rich-Edwards JW, Kleinman KP, Strong EF, Oken E, Gillman MW (2005). Preterm delivery in Boston before and after September 11th, 2001. Epidemiology.

[B26] Ryan MA, Pershyn-Kisor MA, Honner WK, Smith TC, Reed RJ, Gray GC (2001). The Department of Defense Birth Defects Registry: overview of a new surveillance system. Teratology.

[B27] Sever L (2004). National Birth Defects Prevention Network (NBDPN) Guidelines for Conducting Birth Defects Surveillance.

[B28] (1997). ICD-9-CM: International classification of diseases, 9th revision, clinical modification.

[B29] Correa-Villasenor A, Cragan J, Kucik J, O'Leary L, Siffel C, Williams L (2003). The Metropolitan Atlanta Congenital Defects Program: 35 years of birth defects surveillance at the Centers for Disease Control and Prevention. Birth Defects Res A Clin Mol Teratol.

[B30] Sadler TW (2006). Langman's Medical Embryology.

[B31] Mizuno R (2000). The male/female ratio of fetal deaths and births in Japan. Lancet.

[B32] Hansen D, Moller H, Olsen J (1999). Severe periconceptional life events and the sex ratio in offspring: follow up study based on five national registers. BMJ.

[B33] Owen D, Matthews SG (2003). Glucocorticoids and sex-dependent development of brain glucocorticoid and mineralocorticoid receptors. Endocrinology.

[B34] Bruckner T, Catalano R (2007). The sex ratio and age-specific male mortality: Evidence for culling in utero. Am J Hum Biol.

[B35] Catalano R, Bruckner T (2006). Secondary sex ratios and male lifespan: damaged or culled cohorts. Proc Natl Acad Sci USA.

[B36] Creasy RK, Resnik R, Iams JD, Eds (2003). Maternal-Fetal Medicine.

[B37] Smith TC, Smith B, Corbeil TE, Riddle JR, Ryan MA (2004). Self-reported mental health among US military personnel prior and subsequent to the terrorist attacks of September 11, 2001. J Occup Environ Med.

[B38] Benedek DM, Fullerton C, Ursano RJ (2007). First responders: mental health consequences of natural and human-made disasters for public health and public safety workers. Annu Rev Public Health.

[B39] Linton A, Hartzell MC, Peterson MR (2004). Effect of September 11th terrorist attacks on the self-reported health status of Department of Defense health care beneficiaries. Mil Med.

[B40] Glynn LM, Wadhwa PD, Dunkel-Schetter C, Chicz-Demet A, Sandman CA (2001). When stress happens matters: effects of earthquake timing on stress responsivity in pregnancy. Am J Obstet Gynecol.

[B41] Catalano R, Hartig T (2001). Communal bereavement and the incidence of very low birthweight in Sweden. J Health Soc Behav.

[B42] Ryan MA, Smith TC, Smith B, Amoroso P, Boyko EJ, Gray GC, Gackstetter GD, Riddle JR, Wells TS, Gumbs G (2007). Millennium Cohort: enrollment begins a 21-year contribution to understanding the impact of military service. J Clin Epidemiol.

